# *Aspergillus fumigatus* Detection and Risk Factors in Patients with COPD–Bronchiectasis Overlap

**DOI:** 10.3390/ijms19020523

**Published:** 2018-02-09

**Authors:** Stephanie Everaerts, Katrien Lagrou, Kristina Vermeersch, Lieven J. Dupont, Bart M. Vanaudenaerde, Wim Janssens

**Affiliations:** 1 Department of Respiratory Diseases, University Hospitals Leuven, Herestraat 49, B-3000 Leuven, Belgium; stephanie.everaerts@kuleuven.be (S.E.); lieven.dupont@uzleuven.be (L.J.D.); 2 Department of Chronic Diseases, Metabolism & Aging, Laboratory of Respiratory Diseases, KU Leuven, Herestraat 49, B-3000 Leuven, Belgium; kristina.vermeersch@uzleuven.be (K.V.); bart.vanaudenaerde@kuleuven.be (B.M.V.); 3 Department of Laboratory Medicine, University Hospitals Leuven, Herestraat 49, B-3000 Leuven, Belgium; katrien.lagrou@uzleuven.be; 4 Department of Microbiology and Immunology, KU Leuven, Herestraat 49, B-3000 Leuven, Belgium

**Keywords:** sputum galactomannan, *Aspergillus* PCR, *Aspergillus* colonization, corticosteroids

## Abstract

The role of *Aspergillus fumigatus* in the airways of chronic obstructive pulmonary disease (COPD) patients with bronchiectasis is currently unclear. We searched for a sensitive and noninvasive method for *A. fumigatus* detection in the sputum of COPD patients and addressed potential risk factors for its presence. Induced sputum samples of 18 COPD patients and 17 COPD patients with bronchiectasis were analyzed for the presence of *A. fumigatus* by culture, galactomannan detection, and PCR. Of the patients with COPD–bronchiectasis overlap, 23.5% had a positive culture for *A. fumigatus* versus 10.5% of COPD patients without bronchiectasis (*p* = 0.39). The median sputum galactomannan optical density index was significantly higher in patients with COPD and bronchiectasis compared with patients with COPD alone (*p* = 0.026) and ranged between the levels of healthy controls and *A. fumigatus*-colonized cystic fibrosis patients. Both the presence of bronchiectasis and the administration of systemic corticosteroids were associated with sputum galactomannan (*p* = 0.0028 and *p* = 0.0044, respectively) and showed significant interaction (*p* interaction = 0.022). PCR for *Aspergillus* was found to be a less sensitive method, but was critically dependent on the extraction technique. The higher sputum galactomannan levels suggest a more abundant presence of *A. fumigatus* in the airways of patients with COPD–bronchiectasis overlap compared with patients with COPD without bronchiectasis, particularly when systemic corticosteroids are administered.

## 1. Introduction

Chronic obstructive pulmonary disease (COPD) is a highly prevalent disease, characterized by progressive airflow limitation, which is caused by an abnormal inflammatory response to chronic inhalation of irritants, mainly cigarette smoke [1]. Both small airways (obstructive bronchiolitis) and parenchyma (emphysema) are affected. Bronchiectasis is defined by the presence of irreversibly dilated and chronically inflamed bronchi. COPD and bronchiectasis have overlapping clinical features, but the diagnostic criteria are very different. Whereas an irreversible obstructive lung function is obligatory in COPD, bronchiectasis is diagnosed by structural airway abnormalities observed on computed tomography (CT) of the thorax. The combined presence of COPD and bronchiectasis in one patient is not rare. In cohorts of COPD patients, bronchiectasis has been described in 30–60% of patients [2,3,4,5], whereas bronchiectasis cohorts report COPD as the underlying cause in approximately 30% of patients [6,7].The recognition of an overlap is relevant, since these patients not only have a worse prognosis [2,7,8], but their diagnostic and therapeutic approach is also different.

The pathogenesis of bronchiectasis is generally explained by a vicious circle of inflammation, structural damage, impaired mucus clearance, bacterial colonization, and infection [9]. The underlying mechanism for the development of bronchiectasis in a subset of COPD patients remains largely unknown. *Aspergillus fumigatus* is a ubiquitous fungus, which is cleared from the airways by the innate immune system. A damaged airway epithelium, inherited or acquired defects of the innate immune system, and immunosuppressive drugs such as corticosteroids (CS) predispose to aspergillosis. *A. fumigatus* colonization and sensitization associate with bronchiectasis and unfavorable outcomes in asthma and cystic fibrosis (CF) [10,11,12,13]. In COPD, a history of exacerbations and the isolation of pathogenic bacteria in sputum were suggested as potential risk factors for *A. fumigatus* [14]. Although the clinical relevance is not clear, several retrospective studies showed that more than 20% of COPD patients with *A. fumigatus* detected in respiratory samples eventually developed aspergillosis [15,16]. If *A. fumigatus* plays a causal role in the development of bronchiectasis is still a matter of debate.

One major reason for our limited understanding is the lack of sensitive and noninvasive methods to detect *A. fumigatus* in the airways. Mycological culture is not sensitive nor standardized and has led to an underestimation of *A. fumigatus* in the airways [17]. Other methods focus mainly on invasive fungal disease and require invasive procedures, such as immunohistochemistry on airway biopsies or galactomannan detection on bronchoalveolar lavage fluid. Galactomannan is a polysaccharide cell wall component of *Aspergillus* species, which is not present in resting conidia but is secreted by the hyphae during fungal growth. The determination of galactomannan by enzyme immunoassay in serum and bronchoalveolar lavage fluid is validated for diagnosing an invasive *Aspergillus* disease, mainly in immunocompromised patients. Molecular-based techniques such as polymerase chain reaction (PCR) and next-generation sequencing are hampered by complex DNA extraction, high costs, and limited experience with sputum samples. To investigate the role of *A. fumigatus* in patients with COPD, we explored the feasibility of galactomannan detection and *A. fumigatus* PCR on sputum samples.

We assumed a more abundant presence of *A. fumigatus* in the sputum of patients with COPD-bronchiectasis overlap and addressed the role of CS as a potential risk factor.

## 2. Results

### 2.1. Study Group Characteristics

Thirty-six COPD patients were included, of which 17 had bronchiectasis, with a median modified Reiff score of 4. There were no significant differences between the two groups, apart from a significantly lower median value of pack-years (*p* = 0.048) in patients with bronchiectasis. The results are shown in Table 1.

### 2.2. A. fumigatus Antibodies

Patients with COPD-bronchiectasis overlap had a median *A. fumigatus* IgG value of 35.4 mg/L versus 17.4 mg/L in COPD patients without bronchiectasis (*p* = 0.14). On the basis of both ImmunoCAP measurement and skin prick test, three patients without (15.7%) and five patients with bronchiectasis (29.4%) were sensitized to *A. fumigatus* (*p* = 0.37). The results are summarized in Table 2.

### 2.3. Fungal Sputum Culture

Four COPD patients with bronchiectasis (23.5%) had a positive *A. fumigatus* culture versus two COPD patients without bronchiectasis (10.5%) (*p* = 0.39). *Aspergillus nidulans* was found in samples of two COPD–bronchiectasis-overlap patients which were also positive for *A. fumigatus*. When only inocula of 10 μL were considered, three out of six positive cultures were negative. Of the non-inoculated control plates and of the healthy control samples, none grew *A. fumigatus*, but a positive *A. fumigatus* culture was confirmed in all samples of cystic fibrosis patients with previously known *A. fumigatus* colonization.

### 2.4. Sputum Galactomannan

**Figure 1:**
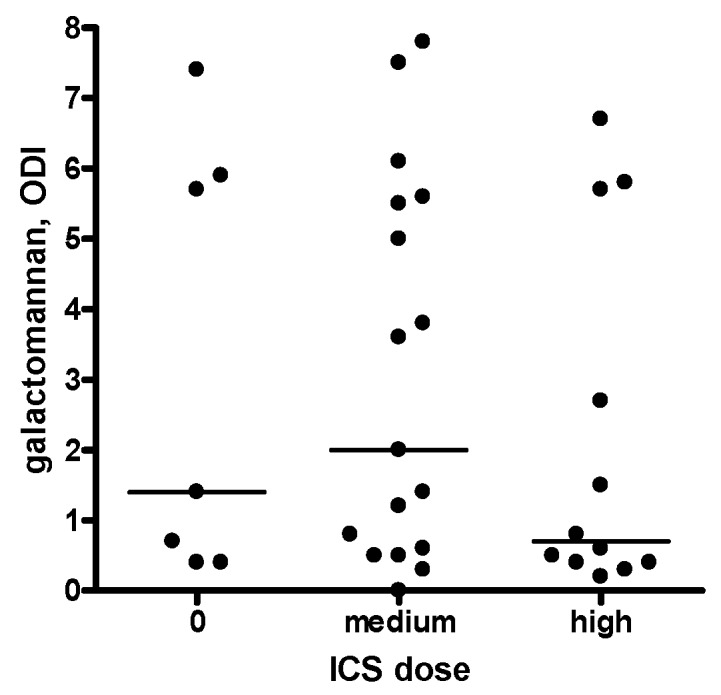
Inhaled corticosteroids and sputum galactomannan. Sputum galactomannan is presented as optical density index. Every dot represents one sample, and the horizontal lines reflect the median values. There was no significant difference in galactomannan values dependent on ICS dose. ODI: optical density index, ICS: inhaled corticosteroids.

**Figure 2:**
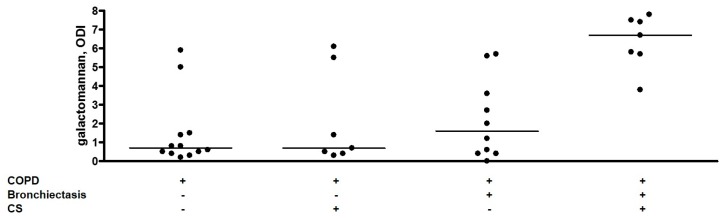
Effect of systemic corticosteroids on sputum galactomannan values. Sputum galactomannan is presented as optical density index. Every dot represents one sample, and the horizontal lines reflect the median values. ODI: optical density index, COPD: chronic obstructive pulmonary disease, CS: systemic corticosteroids in the previous seven days.

**Figure 3:**
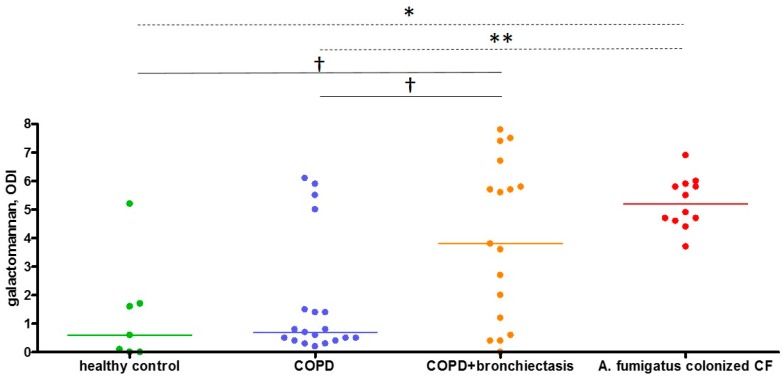
Comparison of COPD sputum galactomannan with healthy and *A. fumigatus*-colonized CF controls. Sputum galactomannan is presented as optical density index. Every dot represents one sample, and the horizontal lines reflect the median values. Kruskal–Wallis test showed an overall significant difference (*p* = 0.0012), with Dunn’s post-hoc comparison for multiple testing showing significant differences between groups, indicated with * *p* < 0.05 and ** *p* < 0.01 (dashed lines). The comparison of COPD + bronchiectasis with the other groups using Mann–Withney U-test showed significant differences between groups, indicated with † *p* < 0.05 (solid lines). ODI: optical density index, COPD: chronic obstructive pulmonary disease, CF: cystic fibrosis.

The median sputum galactomannan ODI was significantly higher in patients with COPD-bronchiectasis overlap compared with patients with only COPD (3.7 versus 0.7, *p* = 0.026) (Table 2). The results of galactomannan measurements in Sputasol, sterile water, and three vials of amoxicillin clavulanate were 0.0, 0.1, and 0.0 respectively. Thirteen sputum samples were retested and had a median galactomannan inter-assay difference of 0.4, with a median coefficient of variation of 9.4% (Supplementary Materials Table S1). The majority of patients was using inhalation corticosteroids (ICS) (Table 1); we found no difference in sputum galactomannan between patients without ICS or with medium or high dose ICS (Figure 1). In contrast to ICS, both bronchiectasis and systemic CS were significantly associated with sputum galactomannan ODI (*p* = 0.0028 and *p* = 0.0044 respectively) in a two-way analysis of variance model. Furthermore, CS showed a stronger association with galactomannan in the patients with COPD–bronchiectasis overlap compared with COPD patients without bronchiectasis (*p* interaction = 0.022). The results are presented in Figure 2 and Table 3. As galactomannan detection on sputum has not been validated, we compared our results to negative and positive control samples and found them to be significantly different (*p* = 0.0012, Figure 3). Moreover, all but one subjects with a positive *A. fumigatus* culture had a sputum galactomannan ODI above 4 (Supplementary Materials Table S2).

### 2.5. Sputum A. fumigatus PCR

When automated DNA extraction was used for *A. fumigatus* real-time PCR, only three samples of COPD patients were found to be positive. All of them had high sputum galactomannan levels. All healthy control samples had a negative PCR result, whereas seven out of twelve CF samples with known *A. fumigatus* colonization had a positive PCR result. Five positive control sputum samples were used to compare automated DNA extraction by EasyMAG and manual extraction by MycXtra^®^. All samples had a positive *A. fumigatus* PCR result after manual DNA extraction compared to only two positive samples after the automated extraction protocol (Table 4). The comparison between sputum galactomannan and *A. fumigatus* PCR is shown in Supplementary Materials Table S2.

## 3. Discussion

Sputum galactomannan levels suggest a higher load of *A. fumigatus* in COPD-bronchiectasis-overlap patients compared to COPD patients without bronchiectasis. The sputum galactomannan values were particularly higher in COPD-bronchiectasis-overlap patients that received systemic CS.

Fungal culture has a low sensitivity, even in proven aspergillosis, which contributes to an underestimation of fungal presence in the airways [18]. In this study, 16.7% of all COPD patients showed *A. fumigatus* in sputum culture. A positive culture was not associated with clinical characteristics or bronchiectasis. Previous studies reported *A. fumigatus* positive cultures in 14–37% of COPD patients [14,19]. In general, culture procedures are not standardized, and the use of different media and methodologies makes it hard to compare different studies. On the basis of a recent report that emphasized the use of larger inocula for fungal culture, we compared sputum volumes of 10 μL and 100 μL [20]. Indeed, we found a higher *A. fumigatus* culture yield by plating 100 μL of the samples. Nevertheless, the lack of standardization and low sensitivity urges the need for other detection methods.

An attractive, alternative method is the detection of galactomannan on sputum samples. On bronchoalveolar lavage fluid, a galactomannan value of 0.5 is suggestive of the presence of *A. fumigatus*, although this cut-off is under debate. Kimura et al. reported 1.2 as the optimal sputum galactomannan cut-off to diagnose invasive aspergillosis in haematological patients [21], whereas Baxter et al. used the 0.5 cut-off for *Aspergillus* positivity in adult CF sputum samples [22]. Recently, the sputum galactomannan results of patients with chronic pulmonary aspergillosis and allergic bronchopulmonary aspergillosis were found to be much higher, with a cut-off above 6.5 for diagnosis [23]. Our study, which used a comparable homogenization of the samples as the latter, found ODI values in the same range. Furthermore, we validated these results with negative and positive controls, which were confirmed by culture and PCR. Despite the higher median galactomannan ODI in COPD patients with bronchiectasis, our study was not able to define an appropriate cut-off. The median coefficient of variation to evaluate inter-assay difference was acceptable, although we are aware that the inter-assay difference was not optimal for some of our samples.

*A. fumigatus* PCR after automated DNA extraction was less sensitive than culture and galactomannan detection on COPD sputum samples. Although the performance is good for clear, liquid samples, our in-house protocol was not suitable for sputum samples, as it does not apply mechanical forces prior to fungal DNA extraction. Manual DNA extraction with MycXtra, which includes bead-beating, performed much better in five additionally collected positive control samples. Since this method required a high sample volume, we were not able to apply this on the other, previously collected study samples. Furthermore, manual extraction is labor-intensive and not suited for large laboratories handling many samples on a daily basis. Any protocol for fungal DNA extraction and PCR on sputum needs further optimization prior to its clinical use.

CS temper the innate immune response, thereby permitting *A. fumigatus* to persist and proliferate. In our study, systemic administration of CS was significantly associated with higher sputum galactomannan values, particularly in patients with COPD–bronchiectasis overlap. These findings contrast with the study of Huerta et al. in which no association was found between *Aspergillus* cultures and oral CS [14]. Differences in the studied population or in the dose of CS administered prior to the analysis may explain these discrepancies. As the large majority of our study subjects on CS received high doses in the context of acute exacerbation (40 mg prednisolone), dose–response relationships could not be studied. Together, our data suggest that systemic CS induce a pronounced and rapid growth of residing *A. fumigatus* in the airways, which may predispose to sensitization, bronchiectasis development, or fungal infection. A local deposition of CS via inhalation may have similar effects. Bafadhel et al. found that COPD patients with *A. fumigatus*-positive culture were on higher doses of inhaled CS compared with culture-negative patients [19]. However, the significance of this association was weak and could not be confirmed by others [14] nor by our analysis.

Although this study does not allow us to attribute causality, our findings support the hypothesis that *A. fumigatus* contributes to the development and/or progression of bronchiectasis in COPD. We recently showed that the sensitization to specific *A. fumigatus* allergens was associated with the presence of bronchiectasis in COPD [5]. Next to an upregulation of the Th2 pathway, *Aspergillus* proteases may play an important role through enhanced mucus production and airway remodeling [24,25]. So far, studies that explored these relationships are solely based on fungal cultures, which may explain some of the inconclusive results. Sputum galactomannan seems to provide a more sensitive marker for *A. fumigatus* detection in the airways of COPD patients. This advantage may serve new longitudinal observational studies or even intervention trials in this area.

## 4. Materials and Methods

### 4.1. Study Design and Subjects

This academic, single-center study included COPD patients prospectively during hospitalization or an outpatient visit. The inclusion criteria were an established diagnosis of COPD based on a post-bronchodilator forced expiratory volume in 1 s (FEV1)/forced vital capacity (FVC) ratio < 0.7, smoking history of at least 10 pack-years, available CT images of the thorax, and an FEV1 ≥ 30% (safety measure for sputum induction). The exclusion criteria were ventilation (mechanical and noninvasive), respiratory diagnosis other than COPD, mycobacterial disease, immunosuppression other than CS, active cancer treatment, history of lung, tracheal, or laryngeal surgery, history of chest radiotherapy, and presence of other inflammatory diseases such as rheumatoid arthritis and inflammatory bowel disease. Investigators were blinded to the results of previous sputum cultures. None of the included patients had a history of allergic or chronic bronchopulmonary aspergillosis, nor invasive *Aspergillus* disease. The presence of bronchiectasis was assessed on CT scans of the lungs. All hospitalized patients received standard therapy for an exacerbation: CS and antibiotics if indicated. They were included after sufficient recovery from the acute event. Oral or intravenous CS and antibiotics administered in the seven days before sputum induction were considered relevant. The study was approved by the local Ethics Committee (Medical Ethical Board of the University Hospitals Leuven, Belgium—M11223) and all patients signed informed consent before enrollment.

### 4.2. Pulmonary Function and Questionnaires

Post-bronchodilator spirometry was measured using standardized equipment (Whole Body Plethysmograph, Vyaire, Vilvoorde, Belgium), according to the American Thoracic Society/European Respiratory Society guidelines [26]. Diffusion capacity was measured by the single-breath carbon monoxide gas transfer method [27]. The results are reported as percentages predicted of reference values. The post-bronchodilator FEV1 was used to classify the patients according to the Global Initiative for Chronic Obstructive Lung Disease (GOLD) classification. The patients completed the modified Medical Research Council (mMRC) breathlessness scale [28], COPD Assessment Test (CAT™) [29], and Saint Georges Respiratory Questionnaire (SGRQ), a self-administered health-related quality of life measure.

### 4.3. CT Thorax

All subjects had a high-resolution CT (HRCT) of the thorax at least one year before enrollment. All patients had inspiratory images with 1 mm slices. Bronchiectasis were defined based on Naidich’s descriptions: bronchoarterial ratio > 1, lack of tapering, and presence of bronchus within 1 cm of the costal pleura or abutting the mediastinal pleura [30]. All images were blinded to the other data and scored using the modified Reiff score, assessesing the number of involved lobes (the lingula considered separately) and the degree of bronchodilation (1 = tubular, 2 = varicose, and 3 = cystic) [31].

### 4.4. Eosinophils, Total IgE, A. fumigatus Sensitization, and A. fumigatus-Specific IgG

Blood samples were collected, and white blood cell count and differentiation were performed by fluorescence flow cytometry (Sysmex XE-5000, Kobe, Japan). Total IgE, IgE against *A. fumigatus* extract, recombinant antigens (rAsp f1-f4 and f6), and *A. fumigatus* IgG were determined by ImmunoCAP fluoroenzyme-immunoassay (Phadia AB, Uppsala, Sweden). The skin prick test with crude *A. fumigatus* extract (ALK, Almere, The Netherlands) was performed according to the guidelines [32].

### 4.5. Sputum Collection, Homogenization, and Culture

In all COPD patients, sputum was induced by inhalation of hypertonic saline (concentration 3%, 4%, and 5% for 5 min each) generated by an Ultra-Neb ultrasonic nebulizer (DeVilbiss, Port Washington, NY, USA) after pretreatment with 400 μg of inhaled salbutamol. The patient was asked to rinse the mouth thoroughly with water and spit the sputum into a collection tube [33]. As negative and positive control samples, induced sputum samples of 7 healthy controls and spontaneous samples of 12 CF patients with known *A. fumigatus* colonization were collected, respectively. All samples were immediately stored at −80 °C. For homogenization, an equal volume of Sputasol^®^ (Oxoid, Thermo Fisher, Hampshire, UK, 1.4% dithiothreitol) was added to each sample. The samples were subsequently incubated at 37 °C for 30 min and shaken every 10 min. Two volumes of each sample, 10 and 100 μL, were inoculated on Sabouraud agar plates (Sabouraud with chloramphenicol (Bio-Rad, Marnes-la-Coquette, France). The culture plates were incubated at 42 °C for 1 day and subsequently at 30 °C for 1 week. For each sample, an additional plate without inoculation was incubated as a control. The growth was checked daily and the colonies were identified by microscopy.

### 4.6. Sputum Galactomannan Assay

Of the homogenized sputum, 300 μL was transferred to a sterile tube. An enzyme immunoassay (Platelia™ *Aspergillus* Ag, Bio-Rad, Marnes-la-Coquette, France) was used to detect galactomannan. The results are expressed as optical density index (ODI), the ratio of the optical density of the sample to the mean cut-off control optical density. To exclude false positivity, galactomannan was determined in Sputasol^®^ and in the sterile water we used to prepare the Sputasol^®^ solution. Furthermore, three vials from different batches of amoxicillin clavulanate were tested, as some of the hospitalized patients received this antibiotic, known to influence galactomannan results in previous reports [34]. Moreover, 13 sputum samples were retested to confirm the results.

### 4.7. Aspergillus PCR on Sputum

*A. fumigatus* real-time PCR was done following an in-house method with excellent performance in European *Aspergillus* PCR Initiative (EAPCRI) evaluations. Nucleic acid extraction was semi-automatically performed with EasyMAG (Biomérieux, Marcy l’ Etoile, France) using NucliSens reagents as recommended by the manufacturer. Briefly, 500 μL of sputum samples was lysed in the presence of magnetic silica beads and subsequently eluted. This method was compared with a manual extraction method using MycXtra^®^ (Myconostica, Cambridge, UK) in five additionally collected CF sputum samples with known *A. fumigatus* colonization. The fungal DNA was extracted as recommended by the manufacturer. Briefly, 1 mL of the homogenized sample was used for extraction, then lysis, bead beating, and purification were performed to remove inhibiting substances with reagents provided by the manufacturer. The nucleic acid extracts where used for real-time PCR (Quantstudio™, Thermo Fisher Life technologies, Carlsbad, CA, USA). The comparative cycle threshold was used to normalize data after running through 45 cycles. The used reagents are shown in the online Supplementary Materials Table S3.

### 4.8. Statistical Analysis

Statistical analysis was performed using GraphPad Prism 4 (GraphPad Software, La Jolla, CA, USA) and SAS software version 9.4 (SAS Institute, Cary, NC, USA). Non-parametric tests were used for analyses. Univariate comparisons were performed by Mann–Withney U-test and presented as median ± interquartile range. The proportions of discrete variables were compared with χ² test and presented as absolute numbers and percentages. The comparison of four groups was performed with the Kruskal–Wallis and post-hoc Dunn’s tests for multiple comparison. A multivariate model was built for two-way analysis of variance to study the association between sputum galactomannan, bronchiectasis, and CS. After performing bivariable models with sputum galactomannan as exposure, potential confounders of the association between sputum galactomannan and bronchiectasis were included in the final model if they (1) changed the estimate of the multivariable model ≥10% or (2) the variable was significantly associated with galactomannan and bronchiectasis. The interaction between bronchiectasis and CS was included in the model. *p*-Values < 0.05 were considered significant in all analyses.

## Figures and Tables

**Table 1:** Characteristics of the study groups.

characteristic	COPD without Bronchiectasis	COPD with Bronchiectasis
Subjects, *n*	19	17
Hospitalized, %	42	65
Age, y	71 (60–75)	68 (63–79)
Male, %	84	88
BMI, kg/m²	25 (20–30)	22 (19–27)
Pack-years *	48 (40–55)	40 (25–45)
FEV1, % pred	51 (39–58)	41 (37–52)
DLCO, % pred	47 (40–57)	44 (26–53)
GOLD		
I/II/III/IV, %	0/53/47/0	6/29/65/0
A/B/C/D, %	21/0/16/63	12/17/6/65
Eosinophils, %	1.4 (0.1–3.1)	2.3 (0.8–4.6)
Eosinophils, μL	200 (0–300)	200 (100–350)
Total IgE, kU/L	83 (16–592)	43 (33–298)
mMRC	2 (1–2)	2 (1.5–3.5)
CAT	14 (12–20)	19 (13–24)
SGRQ	41.9 (30.6–53.4)	54.9 (37.3–66.7)
≥2 exacerbations/y, %	79	71
ICS, %	89	82
Modified Reiff score	NA	4 (2.5–6.5)

Data are presented as *n*, % or median (interquartile range). COPD: chronic obstructive pulmonary disease, y: year, BMI: body mass index, FEV1: forced expiratory volume in 1 s, % pred: percentage predicted, DLCO: diffusion capacity of the lung for carbon monoxide, GOLD: Global initiative for chronic Obstructive Lung Disease stadia, mMRC: modified Medical Research Council breathlessness scale, CAT: COPD Assessment Test, SGRQ: Saint Georges Respiratory Questionnaire, ICS: inhaled corticosteroids. * *p*-value < 0.05. All other variables were not significantly different between the groups.

**Table 2:** *A. fumigatus*-related results.

Test	COPD without Bronchiectasis	COPD with Bronchiectasis	*p*-Value
**Subjects, *n***	19	17	
***A. fumigatus* IgG, mg/L**	17.4 (13.4–37.3)	35.4 (25.5–51.6)	0.14
***A. fumigatus* sensitization, %**	15.7	29.4	0.37
***A. fumigatus* sputum culture, %**	10.5	23.5	0.39
**Sputum galactomannan, ODI**	0.7 (0.4–1.5)	3.7 (0.6–5.7)	**0.026**

Data are presented as *n*, median (interquartile range) or %. COPD: chronic obstructive pulmonary disease, *A. fumigatus*: *Aspergillus fumigatus*, ODI: optical density index. *p*-Value < 0.05 is captured in bold.

**Table 3:** Associations with sputum galactomannan in COPD patients.

**Bivariate Models**		
	**Estimate**	***p*-Value**
CS in COPD without bronchiectasis (*n* = 19)	0.64	0.54
CS in COPD with bronchiectasis (*n* = 17)	4.17	**0.0004**
**Multivariate Model**		
	**Estimate**	***p*-Value**
Bronchiectasis	0.67	**0.0028**
CS	0.57	**0.0044**
Antibiotics	0.37	0.63
Bronchiectasis–CS interaction	3.43	**0.022**

Two bivariate general linear models were built with galactomannan as exposure: one for patients without and one for patients with bronchiectasis showing that CS were only significant associated with galactomannan in patients having bronchiectasis. One multivariate (two-way analysis of variance) general linear model was built for all patients in which systemic corticosteroids and antibiotics administered in the seven days before sputum induction were considered relevant. CS: systemic corticosteroids, COPD: chronic obstructive pulmonary disease, *p*-values < 0.05 are shown in bold.

**Table 4:** Comparison of DNA extraction methods in five samples of cystic fibrosis patients with known *A. fumigatus* colonization.

Subject	Sputum Galactomannan, ODI	PCR after EasyMAG Extraction, Ct	PCR after MycXtra^®^ Extraction, Ct
CF1	4.4	negative	positive, 36.7
CF2	3.7	negative	positive, 32.9
CF3	4.7	positive, 29.3	positive, 26.2
CF4	4.6	positive, 30.7	positive, 30.5
CF5	4.7	negative	positive, 33.2

Sputum galactomannan is presented as optical density index. PCR is expressed as negative or positive with the respective Ct value in case of positivity. CF: cystic fibrosis sample, ODI: optical density index, PCR: polymerase chain reaction, Ct: cycle threshold.

## Supplementary Materials

Supplementary materials can be found at http://www.mdpi.com/1422-0067/19/2/523/s1.
