# Supplementary material for: Aspergillus fumigatus Detection and Risk Factors in Patients with COPD–Bronchiectasis Overlap
Source: Int J Mol Sci. 2018 Feb 9;19(2):523. doi: 10.3390/ijms19020523 (PMC5855745; doi:10.3390/ijms19020523)
Supplement: Supplementary file 1 [file ijms-19-00523-s001.pdf]

SUPPLEMENT

Table S1: inter-assay difference in 13 sputum samples.

Galactomannan 1, ODI	Galactomannan 2, ODI	Difference	CV, %
0.5	0.9	0.4	40.4
0.2	0.2	0	0
5.5	6.3	0.8	9.6
0.3	0.3	0	0
0.7	0.8	0.1	9.4
1.4	1.4	0	0
7.4	6.3	-1.1	11.4
6.7	6.1	-0.6	6.6
3.5	5.2	1.7	27.6
7.8	6.2	-1.6	16.2
2.7	2.5	-0.2	5.4
7.5	6.2	-1.3	13.4
5.6	5.8	0.2	2.5

Galactomannan measurement on same samples of 13 subjects. Galactomannan is presented as optical density index ODI: optical density index, CV: coefficient of variation.

Table S2: Aspergillus sputum culture, sputum galactomannan and PCR per patient

	***A. Fumigatus* culture**	**Sputum galactomannan, ODI**	***A. Fumigatus* PCR, Ct**
COPD 1	-	0.5	-
COPD 2	-	0.2	-
COPD 3	positive	5.5	29.6
COPD 4	-	0.3	-
COPD 5	-	0.7	-
COPD 6	-	1.4	-
COPD 7	-	0.4	-
COPD 8	-	0.5	-
COPD 9	-	1.5	-
COPD 10	-	0.8	-
COPD 11	-	5	-
COPD 12	-	0.3	-
COPD 13	-	5.9	-
COPD 14	-	0.6	-
COPD 15	positive	6.1	27.1
COPD 16	-	0.5	-
COPD 17	-	0.8	-
COPD 18	-	0.4	-
COPD 19	-	1.4	-
COPD+bronchiectasis 1	-	7.4	-
COPD+bronchiectasis 2	positive	6.7	31.1
COPD+bronchiectasis 3	-	3.6	-
COPD+bronchiectasis 4	positive	7.8	-
COPD+bronchiectasis 5	-	2.7	INH
COPD+bronchiectasis 6	positive	7.5	-
COPD+bronchiectasis 7	-	5.6	-*
COPD+bronchiectasis 8	-	0.4	-
COPD+bronchiectasis 9	-	3.8	-
COPD+bronchiectasis 10	-	5.8	-
COPD+bronchiectasis 11	-	5.7	-*
COPD+bronchiectasis 12	-	0.4	-
COPD+bronchiectasis 13	-	0	-
COPD+bronchiectasis 14	-	0.6	-
COPD+bronchiectasis 15	-	5.7	-
COPD+bronchiectasis 16	positive	2	-
COPD+bronchiectasis 17	-	1.2	-
negative control 1	-	1.6	-
negative control 2	-	0	-
negative control 3	-	1.7	-
negative control 4	-	0	-
negative control 5	-	5.2	-
negative control 6	-	0.6	-
negative control 7	-	0.1	-

positive control 1	positive	6.9	35.2
positive control 2	positive	5.8	27.6
positive control 3	positive	4.9	33.3
positive control 4	positive	5.5	-
positive control 5	positive	5.8	29.4
positive control 6	positive	6	-
positive control 7	positive	5.9	30.8
positive control 8	positive	4.4	-
positive control 9	positive	3.7	-
positive control 10	positive	4.7	29.3
positive control 11	positive	4.6	30.7
positive control 12	positive	4.7	-

Galactomannan is presented as optical density index. *A. fumigatus* PCR was performed after automated DNA extraction. If PCR was positive, a Ct value is presented. COPD: chronic obstructive pulmonary disease, ODI: optical density index, PCR: polymerase chain reaction, Ct: cycle threshold, INH: inhibition despite of dilution in second run, *: diluted 1/5 because of inhibition in first run.

Table S3: used reagents for real-time PCR

**Target**	28S rRNA
**Primer, forward**	5'-GCACGTGAAATTGTTGAAAGG-3'
**Primer, reverse**	5'-CAGGCTGGCCGCATTG-3'
**Probe**	FAM-5'-CATTCGTGCCGGTGTACTTCCCCG-3'-TAMRA
**Polymerase**	TaqMan Fast Virus Mix (Life technologies, Carlsbad, USA)
**Temperature program**	95°C – 20 seconds
